# The role of alcohol response phenotypes in the risk for alcohol use disorder

**DOI:** 10.1192/bjo.2019.18

**Published:** 2019-04-22

**Authors:** Andrea C. King, Dingcai Cao, Harriet deWit, Sean J. O'Connor, Deborah S. Hasin

**Affiliations:** Professor, Department of Psychiatry & Behavioral Neuroscience, University of Chicago, USA; Associate Professor, Department of Ophthalmology and Visual Sciences, University of Illinois at Chicago, USA; Professor, Departments of Psychiatry and Biomedical Engineering, Indiana University School of Medicine and Purdue University, USA; Professor, Mailman School of Public Health, Columbia University; College of Physicians and Surgeons; and New York State Psychiatric Institute, USA

**Keywords:** Alcohol, stimulation, sedation, differentiator model, low-level response theory

## Abstract

Heavy alcohol use is pervasive and one of our most significant global health burdens. Early theories posited that certain alcohol response phenotypes, notably low sensitivity to alcohol (‘low-level response’) imparts risk for alcohol use disorder (AUD). However, other theories, and newer measures of subjective alcohol responses, have challenged that contention and argued that high sensitivity to some alcohol effects are equally important for AUD risk. This study presents results of a unique longitudinal study in 294 young adult non-dependent drinkers examined with alcohol and placebo testing in the laboratory at initial enrolment and repeated 5 years later, with regular follow-up intervals assessing AUD (trial registration: http://clinicaltrials.gov/ct2/show/NCT00961792). Findings showed that alcohol sedation was negatively correlated with stimulation across the breath alcohol curve and at initial and re-examination testing. A higher rather than lower alcohol response phenotype was predictive of future AUD. The findings underscore a new understanding of factors increasing vulnerability to AUD.

**Declaration of interest:**

None.

Estimates attribute 4.5% of the global burden of disease and injury to alcohol, with 2.5 million deaths annually caused by harmful alcohol use.^1^ Excessive drinking affects all organ systems, contributing to liver, gastrointestinal, cardiovascular disease, and fetal alcohol spectrum disorder, and also unintentional injuries and accidents. Heavy use of alcohol is pervasive; one-sixth to one-fifth of the European and US populations aged 15 and older report weekly heavy episodic drinking (≥60 g alcohol/occasion).^1,2^ Given the close correspondence between heavy drinking and alcohol use disorders (AUD), a greater understanding of the mechanisms underlying the development of AUD is critical.

Several theories posit that specific alcohol response phenotypes play an important role in the development and maintenance of AUD. The original low-level response theory posits that lesser subjective and behavioural responses to alcohol predict risk for developing AUD, presumably because greater quantities of alcohol must be consumed to achieve a desired effect.^3^ More recent studies indicate that greater responses to alcohol, particularly to its rewarding and stimulating effects perhaps as a result of striatal dopamine release,^4^ is a better predictor of risk for AUD.^5,6^ In an attempt to resolve these discrepant results, an alternative theory, the differentiator model,^7^ posited that AUD risk is marked by both greater stimulatory alcohol effects during the ascending limb of the breath alcohol concentration (BrAC) and lower sedative responses during the descending limb. Our group subsequently refined this model by showing that these alcohol responses simply measured at peak BrAC were predictive of future increases in alcohol consumption and AUD symptoms.^8,9^ Resolving conflicting theories of addiction risk requires sensitive outcome measures, large sample sizes and longitudinal data. Since the time the low-level response theory was formulated, measures of acute subjective effects of alcohol have improved, making it possible to measure alcohol stimulation concurrently with sedation. In our prospective Chicago Social Drinking Project (trial registration: http://clinicaltrials.gov/ct2/show/NCT00961792), we used these measures to assess acute responses to alcohol (versus placebo) over a 5-year period in 294 drinkers varying in risk for AUD. The goal was to examine the long-term relationship between alcohol's stimulant and sedative effects in non-dependent drinkers, and to determine whether higher or lower responses to alcohol predict AUD in early to middle adulthood.

## Method

Participants were healthy young non-alcohol-dependent drinkers (42% female; mean age 25.4, s.d. = 2.9) who were at high or low risk for AUD based on their alcohol consumption patterns. High-risk drinkers (*n* = 208) were defined as those who consumed ≥5 standard drinks (≥4 for women) per occasion 1–4 times/week, >14 units weekly and low-risk, light drinker (*n* = 86) were defined as those who consumed 1–6 drinks/weekly with no/rare heavy drinking. After providing informed consent, participants were individually tested in two 5 h afternoon laboratory sessions in which they consumed either 0.8 g/kg alcohol or placebo in random order under double-blind conditions. They were told the beverage could contain a stimulant, sedative, alcohol or placebo or a combination of two substances. Beverages were consumed in two 5 min intervals separated by a 5 min break and consisted of flavoured drink mix, sucralose-based sugar substitute, water and 16% volume 190-proof ethanol (1% taste mask for placebo), with an average beverage volume of 471 mL. Alcohol doses for women were 85% of those of men to adjust for gender differences in total body water. The University of Chicago Institutional Review Board approved the study.

Breathalyser tests and subjective scales were assessed before initiating beverage consumption and 30, 60 and 120 min after beverage initiation. After 180 min, the participant was provided transportation home with instructions not to drive or operate machinery for 12 h, provided the BrAC was <0.04 g/L with no overt signs of intoxication.

As part of a larger study, the first cohort of 190 participants (104 heavy and 86 light drinkers) were re-tested between 5 and 6 years after initial testing in identical sessions to the initial testing.^10^ The majority (88%) of the 178 deemed eligible for re-examination agreed to participate (86 high-risk and 70 low-risk drinkers). For all participants, follow-up interviews were conducted at 1, 2, 5 and 6 years after initial testing to ascertain the number of AUD symptoms met in the prior year. Those who met mild, moderate or severe AUD by DSM-5 criteria^11^ in two or more of these interviews were deemed AUD+. Through follow-up, AUD+ was evident in 53% (110/208) of high-risk drinkers and 1% (1/86) of low-risk drinkers.

Acute responses to alcohol were assessed using the stimulation and sedation subscales from the modified Biphasic Alcohol Effects Scale (BAES).^12^ Change scores were calculated by subtracting each post-drink measure from the pre-beverage measure for the alcohol session minus the same change score for the placebo session. This calculation was applied across the three session time points corresponding to rising (0.076 g/dL), peak (0.089 g/dL) and declining BrAC (0.073 g/dL).^8,9,13^

The high-risk drinkers in the two cohorts had similar BrAC levels and alcohol responses,^11^ so their initial testing data were combined. Regression analyses determined the association between stimulation and sedation across the BrAC and for the two testing phases, controlling for other risk and sociodemographic factors, including age, gender, ethnicity, education, drinking group and family history of AUD. The frequency of low versus high responders (negative or positive change scores, respectively) was examined over follow-up using a binomial test of people who were AUD+.

## Results

**Fig. 1:**
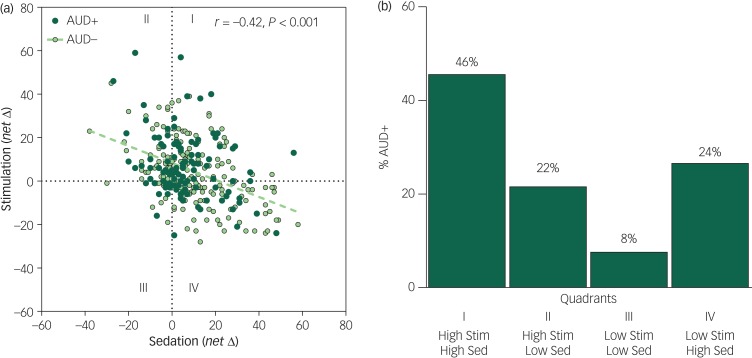
(a) Scatterplots and Pearson correlation between alcohol sedation (*x*-axis) and stimulation (*y*-axis) change scores calculated for the alcohol minus placebo session responses for the whole sample at initial testing at peak breath alcohol concentration (BrAC). (b) Bar graph of the frequency of participants with alcohol use disorder (AUD+) who were initially high- and low-alcohol responders at peak BrAC (change scores from placebo >0 for stimulation, <0 for sedation).

Scores on the BAES sedation and stimulation scales were consistently inversely related at rising, peak and declining BrAC limbs (*r*_s_ ≥ −0.37, *P*_s_<0.001) in both light and heavy drinkers, and at initial and 5-year re-examinations (see Fig. 1(a) for peak BrAC; and supplementary Fig. 1 available at https://doi.org/10.1192/bjo.2019.18 for all BrAC limbs and study phases). Of participants meeting AUD+ during follow-up, at initial testing peak BrAC, very few were low-level alcohol responders (low stimulation and low sedation; 8%, *n* = 9/111; Fig. 1(b), quadrant III), in contrast to 46% who exhibited a high-level alcohol response (*n* = 51/111; Fig. 1(b), quadrant I). This difference in the frequency of low- and high-level responders was also evident during ascending and descending BrAC limbs and persisted through re-examination (2% low-level responders, *n* = 1/47 *v.* 51% high-responders, *n* = 24/47). Thus, drinkers developing AUD were about five times more likely to be high rather than low alcohol responders (*P*<0.001) and this did not change over time.

The majority *n* = 51 (46%; quadrant I in (a) were high responders (high stimulation and high sedation) compared with *n* = 9 (8%) low responders (quadrant III). There were also *n* = 24 (22%; quadrant II) high stimulation responders with low and *n* = 27 (24%; quadrant IV) with low stimulation and high sedation. The AUD+ frequency distribution in the quadrants was highly unlikely (*P*<0.001) resulting from a multinomial distribution with a random chance (i.e. 25% chance for each quadrant), suggesting AUD+ depended on the alcohol stimulation and sedation response pattern. High responders were ~5 times more likely (*P*<0.001 from a binomial test against a 50% chance likelihood) to develop AUD than low responders. Δ, change; Stim, stimulation; Sed, sedation.

## Discussion

Lower alcohol sedation was consistently inversely associated with higher stimulation across the BrAC, for the whole sample and drinker subgroups, and persisted for 5 years. In addition, higher- rather than lower-level responses to alcohol predicted the development of AUD, challenging the conventional notion of the exclusive role of low-level response to alcohol as the key alcohol risk response phenotype. Conflicting theories of the role of alcohol response in the risk for future AUD may have resulted from inconsistencies in examining alcohol effects relative to placebo, and lack of attention to the higher stimulation that is associated with AUD.^8–10^

Our findings from the most extensive repeated alcohol challenge study to date warrant a new understanding of the risk for AUD. Sensitivity to both the stimulating and sedating effects of alcohol may underlie its reinforcing properties^4^ and foster heavy drinking and development of AUD. Early identification of this alcohol response phenotype may provide information for interventions that could reduce the burden of heavy drinking and AUD in society.

## Supplementary material

For supplementary material accompanying this paper visit http://dx.doi.org/10.1192/bjo.2019.18. click here to view supplementary material
