# Supplementary material for: The role of alcohol response phenotypes in the risk for alcohol use disorder
Source: BJPsych Open. 2019 Apr 22;5(3):e38. doi: 10.1192/bjo.2019.18 (PMC6520530; doi:10.1192/bjo.2019.18)
Supplement: Supplementary file 1 [file S2056472419000188sup001.docx]

**Supplemental materials: Figure**

**Legend**

**Scatterplots and Pearson correlations between alcohol sedation (x-axis) and stimulation (y-axis) change scores calculated for the alcohol minus placebo session responses for the whole sample at initial testing and the 5-year re-examination phases**.

Dark circles are AUD+ and light circles are AUD-. (1a) ascending limb of the BrAC at 30 minutes post ingestion, (1b) peak BrAC at 60 minutes post ingestion, and (1c) declining limb of the BrAC at 120 minutes post ingestion. Among participants with AUD+ (N = 111, solid circles), 51(46%) were initially high responders at peak BrAC (Figure 1b, quadrant I, 1b), 9(8%) were low responders (quadrant III), 24 (22%) were high stimulation but low sedation responders (quadrant II), and 27(24%) were low stimulation and high sedation responders (quadrant IV). The AUD+ frequency distribution in the quadrants was highly unlikely (*p* < 0.001) resulting from a multinomial distribution with a random chance (i.e. 25% chance for each quadrant), suggesting AUD+ depended on alcohol stimulation and sedation response pattern. High responders (quadrant I) were ~5 times more likely (*p* < 0.001 from a binomial test against a 50% chance likelihood) to develop AUD than low responders (quadrant III). These responses persisted through re-examination (2%, n=1/47 low level responders versus 51%, n=24/47high responders*, p* <0.001).

**Supplemental Figure**
